# Maternal and infant immunity against *Bordetella pertussis,* Norway, 2020 to 2023

**DOI:** 10.2807/1560-7917.ES.2025.30.50.2500450

**Published:** 2025-12-18

**Authors:** Margrethe Greve-Isdahl, Thea Kristine Rogne Møller, Terese Bekkevold, Marta Natalia Baranowska-Hustad, Cathinka Halle Julin, Ida Laake, Are Stuwitz Berg, Preben Aavitsland, Per Kristian Knudsen, Audun Aase, Ketil Størdal

**Affiliations:** 1 Norwegian Institute of Public Health, Oslo, Norway; 2 University of Oslo, Oslo, Norway; 3 Norwegian Medical Products Agency, Oslo, Norway; 4 Oslo University hospital, Clinical Trials Unit, Oslo, Norway; 5 University of Bergen, Bergen, Norway; 6 Oslo University Hospital, Department of Paediatric Research, Oslo, Norway

**Keywords:** Whooping cough, antibodies, maternal vaccination, childhood immunisation programme

## Abstract

**BACKGROUND:**

Pertussis remains a serious threat to young infants. In Norway, infants receive an acellular pertussis vaccine (aP) according to a 2 + 1 schedule at 3, 5 and 12 months of age, delivered as a hexavalent vaccine.

**AIM:**

We aimed to study susceptibility to pertussis in mothers and infants to guide decisions regarding vaccination in pregnancy.

**METHODS:**

In this prospective observational study, we included 366 mother/infant pairs during 2020–2023, collecting blood samples from mothers in late pregnancy, cord blood at delivery and from infants before their first and after their third vaccine dose. We retrieved health registry data on vaccination and pregnancy-related information. IgG antibody levels against pertussis-antigens, diphtheria and tetanus were measured using a multiplex immunoassay.

**RESULTS:**

Of the pregnant women, 48% (174/366) had low levels of antibodies against pertussis toxin (PT) defined as below 5 IU/mL. Maternal antibodies declined in infants from birth until first vaccination, leaving 72% (154/215) of infants with anti-PT IgG levels below 5 IU/mL. All infants responded well to vaccination and we found no evidence of blunting from high levels (> 40 IU/mL) of maternal antibodies against PT. Infants of mothers who received an aP-containing booster vaccine within 2 years before pregnancy displayed low anti-PT IgG levels, with 58% (15/26) having levels below 5 IU/mL.

**CONCLUSION:**

A high proportion of pregnant women and their infants under 3 months of age had low anti-PT antibody levels, indicating high susceptibility to pertussis. The results support the introduction of vaccination against pertussis during pregnancy in Norway.

**Key public health message**

**What did you want to address in this study and why?**

Whooping cough is a vaccine-preventable disease, caused by the bacterium *Bordetella pertussis*, and can cause severe respiratory disease and even death in infants. We wanted to investigate the protection against whooping cough in pregnant women and their infants in Norway and measured antibodies to evaluate their susceptibility towards this infection.

**What have we learnt from this study?**

Forty-eight percent (174/366) of pregnant women and 72% (154/215) of their infants 2–3 months of age had low antibody levels against whooping cough and are considered highly susceptible to the infection. This was true even for women who had received booster doses within 2 years before pregnancy start and had received childhood vaccines recommended at the time.

**What are the implications of your findings for public health?**

Booster vaccines before pregnancy start will not provide sufficient antibody levels in infants to protect them against whooping cough. The results demonstrate the necessity of efficient vaccination programmes against whooping cough during pregnancy.

## Introduction

Pertussis (whooping cough), caused by the bacterium *Bordetella pertussis*, remains a health threat for infants despite widespread vaccination, causing an estimated 160,700 annual deaths globally in 2014 among children below the age of 5 years [1]. Over the last decades, there has been a concerning resurgence of pertussis in high-income countries, marked by hospitalisations and even deaths in infants [2-4]. Greater awareness and more sensitive diagnostic methods may partly explain this trend, but the switch from whole cell pertussis vaccines (wP) to acellular pertussis vaccines (aP) is considered a main contributor [2]. Therefore, emphasis on implementing additional strategies using the currently available aP vaccines to prevent pertussis, particularly among infants, is essential.

The national childhood immunisation programme in Norway is voluntary, government-funded and provided by public health nurses in municipal child health clinics. In Norway, wP was used in the infant immunisation schedule from 1952 until it was replaced by aP in 1998. The current infant schedule consists of a two-dose primary series at 3 and 5 months with a booster at 12 months (2 + 1 schedule), given as a hexavalent vaccine against diphtheria, tetanus, pertussis, poliovirus, *Haemophilus influenzae* type b and hepatitis B (DTaP/IPV/Hib/HepB). The vaccination coverage is consistently higher than 95% for three doses among infants [5]. In addition, school-age boosters are offered at age 7 and 15 years. Adults are likewise recommended boosters every 10 years, although the awareness of this recommendation is low and adherence is assumed to be variable [6].

Despite the high childhood vaccination coverage at all measured time points (2, 9 and 16 years of age), the registered incidence of pertussis in Norway is among the highest in Europe [7], with a reported incidence of 37–48 cases per 100,000 population annually in 2015–2019 (see Supplementary Figure S1 for incidence rates for reported cases of pertussis). An unusually low incidence of pertussis was observed in all European Union/European Economic Area countries during the COVID-19 pandemic [5], and Norway recorded merely 0.7–0.8 cases per 100,000 population in 2021 and 2022. The low incidence raised concerns of a potential outbreak, including severe disease among infants due to immunity debt resulting from reduced pathogen circulation and infections. This accelerated the review of vaccination policies in several European countries, including Norway, experiencing large pertussis outbreaks in 2024 [5].

Vaccination in pregnancy, inducing maternal antibody transfer to the foetus, is a feasible approach to protect the neonate [8,9]. While maternal pertussis vaccination raises infants' antibody levels, there are concerns that blunting (negative interference with vaccine immune responses) could increase the risk of pertussis in infants and children at a later age [10-13]. The risk of blunted infant vaccine responses should therefore be considered when planning maternal vaccination programmes. Prior to May 2024, maternal vaccination against pertussis was not offered in the national immunisation programme in Norway.

The aim of this study is to investigate susceptibility to pertussis among Norwegian mothers and infants by measuring antibody levels in serum samples from pregnant women and their offspring and study potential blunting of infant vaccine responses from pre-existing maternal antibodies. The results intended to guide decisions on maternal vaccination against pertussis.

## Material and methods

**Figure 1:**
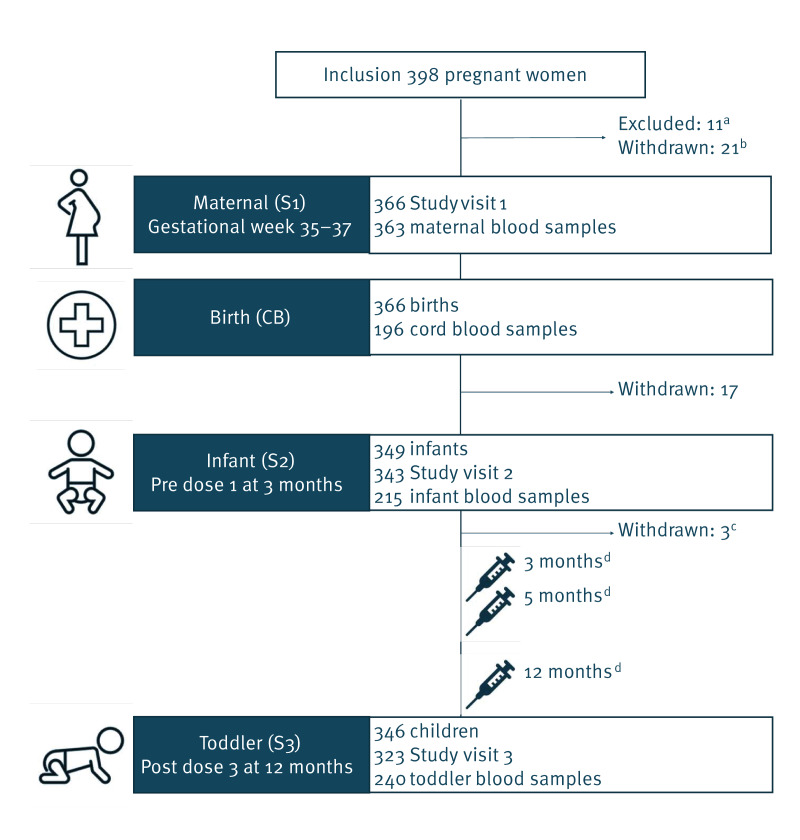
Study outline, Norway, February 2020–October 2023

CB: cord blood; S1: Study visit 1 maternal visit in late pregnancy; S2: Study visit 2 infant age 2–3 months, pre-vaccination with first acellular pertussis vaccine (aP); S3: Study visit 3 infant age 13–15 months, post 12-month booster with aP.

^a^ Excluded due to delivery before Study visit 1 or missing consent from the other parent.

^b^ Withdrawn before Study visit 1.

^c^ One infant died at 6 months of age from causes unrelated to pertussis. The other two withdrew.

^d^ Hexavalent vaccine (diphtheria, tetanus, acellular pertussis, polio, *Haemophilus influenzae* type b, hepatitis B (DTaP/IPV/Hib/HepB), provided at municipal child health clinic.

In this single centre observational prospective study, we collected blood samples and data from a cohort of women, recruited in late pregnancy, and their infants. The mother/infant pairs were followed until blood samples were collected after the infants’ received their first aP-containing hexavalent booster dose at 12 months of age (Figure 1).

### Population

Between February 2020 and March 2022, we recruited a total of 398 pregnant women from the catchment area of Oslo University Hospital who attended the routine ultrasound examination in their second trimester. Participants were eligible if they were born before 1998, had a singleton pregnancy, were considered healthy and stated that they had received childhood vaccines according to the national immunisation programme in Norway at the time. Participants with severe complications during pregnancy and conditions or treatment compromising immunity were not eligible. Eleven women were excluded after recruitment due to delivery before the first study visit or missing consent from the other parent and 21 withdrew before the first study visit, leaving 366 pregnant women and their infants in the study (Figure 1). The final study visit took place in October 2023.

### Data collection

Participants were invited to three study visits at Oslo University Hospital. The first study visit (S1) was in late pregnancy (from gestational week (GW) 35 to 37), the second visit (S2) was before the infant received the first hexavalent vaccine dose at 3 months of age (pre-vaccination), and the last visit (S3) was at 1 to 3 months after the 12-month booster. The hexavalent vaccine administered during the study period was Infanrix Hexa (GlaxoSmithKline, Rixenart, Belgium). Blood samples were collected from the mother during the first study visit, and from the infant at birth (cord blood) and at Study visits 2 and 3 (Figure 1). Questionnaire information was collected during study visits or shortly after and used to collect baseline characteristics, information on the mother’s and infant’s health and identify possible exposure to pertussis. All vaccines were delivered at municipal child health clinics at routine consultations.

We calculated a sample size of 320 mother/infant pairs to have power to estimate vaccine responses in infants with a 95% precision and identify any blunting effect of pre-existing high antibody levels in the mothers on their infants’ vaccine responses. Accounting for a 20% drop-out proportion, the aim was to include 400 pregnant women.

### Health registry data

Using the national personal identity number unique for each participant, data from health registries were linked to laboratory and questionnaire results.

Vaccination data for mothers and children were retrieved from the Norwegian Immunisation Registry (SYSVAK), which was established for five regions in Norway in 1976 and nationally in 1995 [14]. Registration of childhood vaccines is mandatory by law. Registration of adult vaccines may be less complete but became mandatory in 2011.

Information regarding pregnancy, birth and the newborn was retrieved from The Medical Birth Registry of Norway (MBRN). The MBRN receives mandatory notifications for all births from GW 22 or with infant birth weight of at least 500 g [15].

### Laboratory methods

IgG antibody levels against the vaccine components from participants’ serum were analysed by an in-house multiplex immunoassay (MIA) adopted from van Gageldonk et al. [16,17]. Pertussis toxin (PT), pertactin (PRN) and tetanus toxin (TT) (all from List Biological laboratories, Campbell, United States (US)), filamentous haemagglutinin (FHA) (Sigma-Aldrich, Merck Life Science, Darmstadt, Germany), and diphtheria toxoid (DT) (Statens Serum Institute, Copenhagen, Denmark) were coupled to carboxylated microspheres (MagPlex, Bio-Rad, Hercules, US). The antigen-coupled beads were mixed with diluted serum (1:200 and 1:1,000), fourfold dilution of standard serum, three positive controls and one negative control, all in duplicate. After incubation and washing procedures, secondary antibody goat anti-human IgG PE (P8047, Sigma-Aldrich, Merck Life Science) was added. Analyses were conducted on a BioPlexTM System 200 instrument and the BioPlex manager software version 6.2 (Bio-Rad). At these assay conditions, the lower limit of quantification for anti-PT IgG is less than 0.1 IU/mL.

Pertussis toxin is the only *B. pertussis* specific antigen included in all available aP vaccines. There is no established correlate for protection against pertussis, but individuals with antibody levels against pertussis toxin (anti-PT IgG) below 5 IU/mL are considered to have low levels of protection and to be susceptible to infection [18,19]. Anti-PT IgG above 40 IU/mL was used to indicate high levels of antibodies against pertussis, comparable to maternal antibody levels in studies where mothers were vaccinated in pregnancy [12,20]. For IgG against PRN and FHA, which are not specific to *B. pertussis*, no cut-off levels have been suggested. Antibody levels against DT and TT were included in the multiplex immunoassay and analysed for a more comprehensive understanding of vaccine immunity as pertussis vaccines are always combined with vaccines against diphtheria and tetanus. For IgG antibodies against DT and TT, levels above 1.0 IU/mL are considered highly protective, while values below 0.015 IU/mL for anti-DT IgG and 0.1 IU/mL for anti-TT IgG are considered below the protective level [19].

For the blunting analysis, only results from children sampled from 10 to 90 days after their hexavalent booster were included. Using the categorisation for antibody levels described above, we compared antibody levels against PT, DT and TT in infants after the 12-month booster by exposure to low, medium or high maternal antibody levels in late pregnancy.

### Statistical analyses

We compared antibody levels between groups of infants or groups of mothers using a Mann–Whitney U test. To compare antibody levels at Study visit 3 (after the 12-month booster) to antibody levels at Study visit 2 (pre-vaccination), we applied a Wilcoxon matched-pairs signed-rank test. Non-parametric tests were used since antibody levels were not normally distributed. A two-tailed p value < 0.05 was considered significant in all analyses.

All analyses were done using Stata version 17 (StataCorp, College Station, US). Figures were created using Stata or GraphPad Prism version 9.0.0 (GraphPad Software, Boston, US, www.graphpad.com).

## Results

The 366 mother/infant pairs attended a total of 1,032 study visits, producing 1,014 blood samples (Figure 1).

Median maternal age was 33.2 years, and 56.8% (208/366) of the women were nulliparous (Table 1). All included mothers stated that they had received all vaccines according to the Norwegian childhood immunisation programme at the time, and vaccination data were available in SYSVAK for 344 of the women (92.4%); at least one dose of wP was recorded in the first year of life for all 344. The majority of mothers had a registered adult booster vaccine with aP (n = 212, 58%), ranging 15 days to 18.5 years (median 5.5 years) before the start of pregnancy. Four women had received a booster vaccine against diphtheria, tetanus, pertussis and polio (Tdap-IPV) booster during the current pregnancy for unknown reasons.

**Table 1:** Baseline characteristics of the study population, Norway, February 2020–October 2023 (n = 366)

Characteristics	n	%
Mothers (total: n = 366)		
Study visit 1 attended (late pregnancy)	366	100
Mothers with blood sample from Study visit 1	363	99.2
Age at delivery in years, median (IQR)	33.2 (30.7–36.0)	NA
Parity		
Nulliparous	208	56.8
Para 1	126	34.4
Para 2 +	32	8.7
Mothers’ childhood wP vaccination^a^ status in SYSVAK		
≥ 1 dose (range of registration period in years 1976–1997)	344	92.4
≥ 3 doses (range of registration period in years 1976–1997)	332	90.7
Mothers’ aP vaccine^a^ status in SYSVAK		
Mothers with aP vaccine before pregnancy start	212	57.9
Mothers with no recorded aP vaccine	154	42.1
Time before pregnancy start for aP vaccine^a^		
< 2 years	34	9.3
2–5 years	62	16.9
5–10 years	99	27.0
> 10 years	17	4.6
Vaccination during pregnancy		
Influenza^b^ vaccination^a^ during pregnancy	210	57.4
COVID-19^b^ vaccination^a^ during pregnancy	130	35.5
Pertussis (aP) vaccination^a^ during pregnancy	4	1.1
Infants (total: n = 366)		
Male	165	45.1
Female	201	54.9
Total infants providing blood samples	325	88.8
Total infants attending study visits	355	97.0
Cord blood samples collected	196	53.6
Study visit 2 attended	343	NA
Study visit 2 blood sample collected (total: n = 343)	215	62.7
Study visit 3 attended	323	NA
Study visit 3 blood sample collected (total: n = 323)	240	74.3
Infants with complete sample sets from all time points	99	27.0
Gestational age at birth		
Median (IQR) weeks+days	40+1 (39+0–41+0)	NA
< 37 weeks	6	1.6
37–41 weeks	264	72.1
> 41 weeks	96	26.2
Birth weight		
Median (IQR) grams	3,560 (3,240–3,900)	NA
< 3,000	36	9.8
3,000–3,999	263	71.9
≥ 4,000	67	18.3
Age (days)	Median	IQR
At Study visit 2	74	72–78
At Study visit 3	413	401–430
At first hexavalent vaccine dose with aP^a^	93	90–96
At 12-month booster (third hexavalent vaccine) dose with aP^a^	370	362–381

aP: acellular pertussis vaccine (aP-containing hexavalent vaccine also including vaccines against diphtheria, tetanus, poliovirus, *Haemophilus influenzae* type b and hepatitis B (DTaP/IPV/Hib/HepB)); IQR: interquartile range; SYSVAK: Norwegian Immunisation Registry; wP: whole cell pertussis vaccine.

Study visit 1: maternal visit in late pregnancy; Study visit 2: infant age 2–3 months, pre-vaccination with first aP; Study visit 3: infant age 13–15 months, post 12-month booster with aP.

^a^ Vaccination data from Norwegian Immunisation Registry SYSVAK.

^b^ Not all participants may have been recommended an influenza and/or COVID-19 vaccines, as influenza vaccination is recommended only during the influenza season for pregnant women in the second and third trimester, or first trimester if in a risk group or healthcare worker. A recommendation for COVID-19 vaccination in pregnancy (all trimesters) was issued in midst of the study on August 18, 2021.

The infants in the study were born between 24 June 2020 and 4 September 2022. Most infants were born at term (GW 37–41, 72.1%) or post term (GW > 41, 26.2%). Six infants were born as late preterm infants (35–36 weeks). No known exposure to pertussis or reported cough of long duration was declared by parents or legal guardians in the questionnaires. All children received their first hexavalent vaccine after Study visit 2 and their 12-month booster before Study visit 3 (Table 1).

### Maternal antibodies in mothers and infants before first vaccine dose

**Figure 2:**
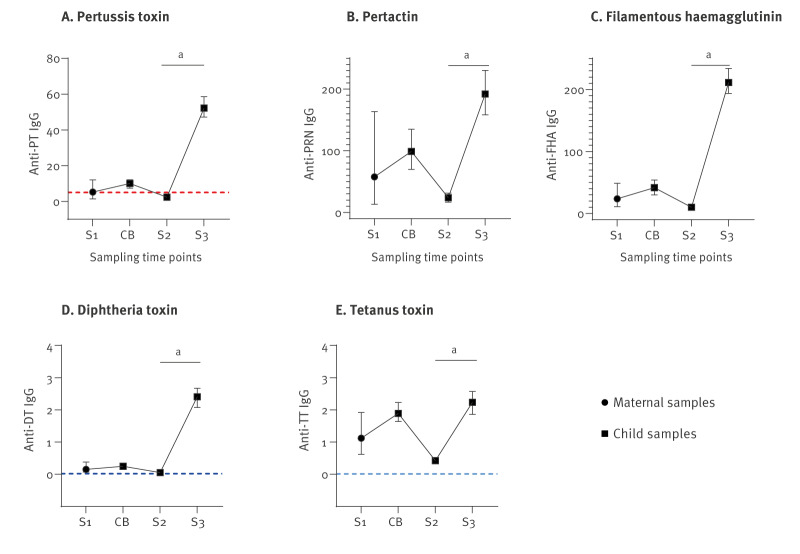
Median IgG antibody levels (IU/mL) for pertussis toxin, pertactin, filamentous haemagglutinin, diphtheria toxin and tetanus toxin obtained from mother or infant by sampling time point, Norway, February 2020–October 2023

In late pregnancy, mothers had low levels of anti-PT IgG (Figure 2), with a median value of 5.3 IU/mL (Table 2), and 48% (174/366) had values below 5 IU/mL. Only 18 mothers (5%, 18/366) had anti-PT IgG above 40 IU/mL, including two of the four mothers who had received a booster vaccine in pregnancy. Two mothers had anti-PT IgG levels above 100 IU/mL, neither of them were vaccinated during pregnancy. Anti-PT IgG was moderately correlated with antibody levels towards the other pertussis antigens (see Supplementary Table S1 for antibody levels in maternal pregnancy samples and Supplementary Figure S2A, 2B for anti-PT IgG in mothers with corresponding IgG-levels against other antigens). For DT and TT, most mothers had protective antibody levels (Table 2). Thirteen mothers with anti-DT IgG below 0.015 IU/mL and nine mothers with anti-TT IgG below 0.1 IU/mL also had anti-PT IgG below 5 IU/mL (see Supplementary Figure S2C, 2D for anti-PT IgG in mothers with corresponding IgG-levels against other antigens). Further analysis did not indicate any association with maternal age, parity or infant birth weight (see Supplementary Figure S3 for median anti-PT IgG-levels by maternal age, parity and infant birth weight).

CB: cord blood; S1: Study visit 1 (maternal visit in late pregnancy); S2: Study visit 2 (infant age 2–3 months, pre-vaccination with first acellular pertussis vaccine (aP)); S3: Study visit 3 (infant age 13–15 months, post 12-month booster with aP).

Red dashed line at anti-PT IgG = 5 IU/mL (Panel A); blue dashed line indicates anti-DT IgG = 0.015 IU/mL (Panel D); light blue dashed line indicates anti-TT IgG = 0.1 IU/mL (Panel E).

^a^ Indicates statistical significance.

**Table 2:** Median IgG antibody levels (IU/mL) for pertussis toxin, diphtheria toxin, tetanus toxin, pertactin and filamentous haemagglutinin at each of the four sampling time points, Norway, February 2020–October 2023

Study visit	Mother		Child													
	Pregnancy		Birth cord blood		Pre-vaccination		Post 12-month booster		Post 12-month booster Grouped according to time after hexavalent booster (DTaP/IPV/Hib/HepB)							
	S1		CB		S2		S3 (All)		S3^a^							
IgG antibodies (IU/mL)	Median	IQR	Median	IQR	Median	IQR	Median	IQR	10–90 days		10–29 days		30–90 days		> 90 days	
									Median	IQR	Median	IQR	Median	IQR	Median	IQR
Pertussis toxin	5.3	1.4–12.1	10.0	2.7–21.9	2.4	0.6–5.7	52.2	29.3–95.5	55.5	31.6–97.9	75.7	36.3–103.2	53.4	31.5–96.9	24.9	10.4–42.6
Diphtheria toxin,	0.15	0.07–0.38	0.25	0.09–0.64	0.05	0.02–0.12	2.41	1.39–3.87	2.60	1.55–3.96	3.54	2.34–4.68	2.51	1.46–3.79	0.55	0.37–1.02
Tetanus toxin	1.12	0.62–1.92	1.89	1.12–2.97	0.42	0.24–0.72	2.23	1.17–4.29	2.36	1.36–4.45	2.31	1.33–4.70	2.36	1.36–4.30	0.85	0.64–2.14
Pertactin	58.2	13.3–165.2	98.9	19.5–268.7	23.9	4.9–59.7	192.0	92.6–385.7	NA		NA		NA		NA	
Filamentous haemagglutinin	23.7	10.9–48.7	41.6	17.7–84.7	10.1	4.6–22.6	211.3	118.7–341.6	NA		NA		NA		NA	
Total samples (n)	363		196		215		240		215		32		183		22	

CB: cord blood; DTaP/IPV/Hib/HepB: acellular pertussis-containing hexavalent vaccine includes vaccines against diphtheria, tetanus, poliovirus, *Haemophilus influenzae* type b and hepatitis B; IQR: interquartile range; S1: Study visit 1 (maternal visit in late pregnancy); S2: Study visit 2 (infant age 2–3 months, pre-vaccination with first acellular pertussis vaccine (aP)); S3: Study visit 3 (infant age 13–15 months, post 12-month booster with aP).

^a^ Three infants were sampled less than 10 days after the 12-month booster and were excluded in the blunting analysis.

The transplacental IgG transfer from mothers to neonates was high for all studied antibodies (Table 2, Figure 2), with median ratios ranging between 1.5 and 1.8, thus providing the neonates with higher antibody levels than their mothers (see Supplementary Table S2 for placental antibody transfer ratios). Still, 36% (70/196) of neonates had anti-PT IgG below 5 IU/mL in cord blood.

The antibody levels declined in infant sera from birth to pre-vaccination at 2 to 3 months of age (Table 2, Figure 2), and a high proportion of infants (72%, 154/215) had anti-PT IgG below 5 IU/mL indicating low protection. Levels of anti-PT correlated moderately with PRN and FHA antibodies in infants at 2 to 3 months of age (see Supplementary Figure S4 for anti-PT IgG in infants at Study visit 2 with corresponding IgG-levels against other antigens), which was also demonstrated in late pregnancy.

### Vaccine responses in infants and blunting

After the 12-month booster, all but three children achieved anti-PT IgG levels above 5 IU/mL, with a median level of 52.2 IU/mL (Table 2) and a significant increase compared with pre-vaccination levels (p < 0.001) (Figure 2). However, not all blood samples were collected at the recommended interval after the 12-month booster: three children were sampled fewer than 10 days after the booster, not leaving enough time to generate a detectable vaccine response. In addition, 22 children were sampled more than 90 days after, and these displayed significantly lower IgG levels for PT than in sera collected 10 to 90 days after vaccination (p < 0.01, Supplementary Figure S5: median anti-PT IgG at Study visit 3 grouped by days after 12-month aP-booster). Children with samples collected outside the 10–90-day window, including all three with anti-PT below 5 IU/mL, were excluded from the blunting analysis (see Supplementary Figure S6 for individual anti-PT IgG at Study visit 3 by days after 12-month aP-booster).

**Figure 3:**
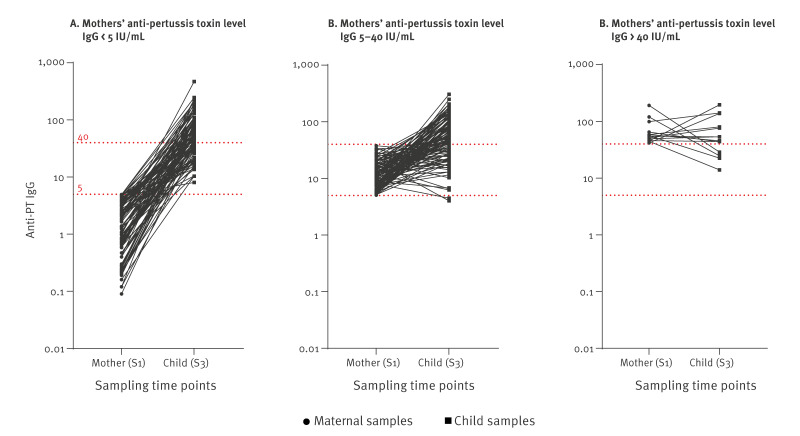
Anti-pertussis toxin levels, log transformed, in mothers in late pregnancy at Study visit 1 and in their children after receipt of 12-month booster (Study visit 3), Norway, February 2020–October 2023

In the blunting analysis, we included the 215 mother-infant pairs where the final blood sample was collected between 10 and 90 days after the 12-month booster. The median child antibody levels for anti-PT IgG were not significantly different when comparing children of mothers with maternal anti-PT IgG levels below 5 IU/mL (median 52.3 IU/mL) with children of mothers with intermediate maternal levels (median 63.2 IU/mL, p = 0.66) or with high maternal anti-PT IgG levels higher than 40 IU/mL (median 49.3 IU/mL, p = 0.76) (Figure 3). We also found no blunting effect of anti-DT IgG or anti-TT IgG (see Supplementary Table S3 for information on blunting analysis).

Dotted red lines indicate anti-pertussis toxin (PT) IgG = 5 IU/mL, log transformed and anti-PT IgG = 40 IU/mL, log transformed.

### Mothers’ prior acellular pertussis vaccination and antibody levels

**Figure 4:**
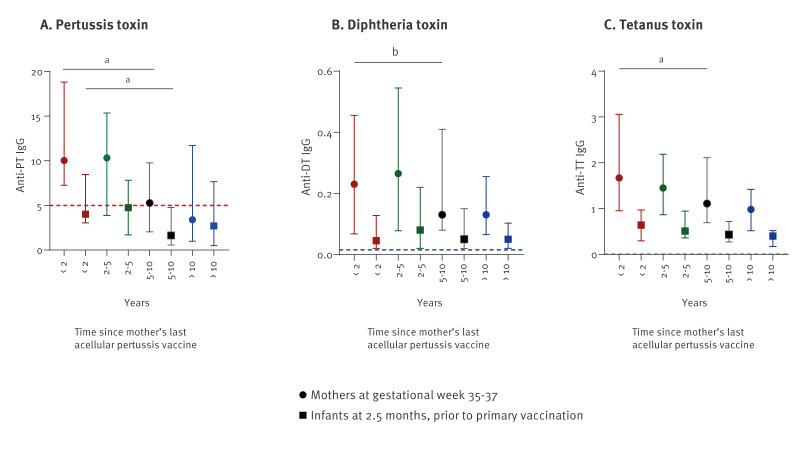
Median IgG antibody levels (IU/mL) for pertussis toxin, diphtheria toxin and tetanus toxin in mothers in late pregnancy and infants pre-vaccination at 2–3 months of age according to time since mothers’ last registered acellular pertussis vaccine, Norway, February 2020–October 2023

DT: diphtheria toxin; PT: pertussis toxin; TT: tetanus toxin.

^a^ Indicates statistical significance

^b^ indicates not significant.

Among the 212 mothers who received an adult aP-containing vaccine before pregnancy start (mainly TdaP-IPV), antibody levels for PT and TT were lower in mothers vaccinated 5–10 years and more than 10 years before pregnancy compared with mothers who had received a vaccine within the 2 years before pregnancy (p < 0.05 for both PT and TT), but not for DT (p ≥ 0.24) (Figure 4). For DT and TT, observed median antibody levels in late pregnancy were considered protective. The median PT-level was at 5.3 IU/mL and below if more than 5 years had elapsed since last aP. Maternal anti-PT levels declined with time since last aP before pregnancy, but not according to calendar time (Supplementary Figure S7: mothers’ anti-PT IgG by time since last aP and calendar date for sampling). Among infants at pre-vaccination, we found a median anti-PT IgG below 5 IU/mL in all groups, i.e. even when mothers had received an aP-vaccine within the 2 years before pregnancy, 15 of 26 infants had anti-PT IgG levels below 5 IU/mL (Figure 4). The anti-PT IgG-levels declined significantly in infants with time since their mothers’ last registered aP (p < 0.01).

Red indicates mothers’ last acellular pertussis vaccine (aP) less than 2 years before pregnancy start. Green indicates mothers’ last aP 2–5 years before pregnancy start. Black indicates mothers’ last aP 5–10 years before pregnancy start. Blue indicates mothers’ last aP more than 10 years before pregnancy start.

Red dashed line at anti-PT IgG = 5 IU/mL (Panel A); blue dashed line indicates anti-DT IgG = 0.015 IU/mL (Panel B); light blue dashed line indicates anti-TT IgG = 0.1 IU/mL (Panel C).

## Discussion

In this prospective cohort study, we demonstrate that a significant proportion of pregnant women had low levels of antibodies against pertussis in late pregnancy, despite adherence to recommended childhood vaccinations and receipt of adult booster doses, in contrast to protection against diphtheria and tetanus. This leaves both mothers and their infants susceptible to pertussis, with 72% of infants having low anti-PT IgG levels below 5 IU/mL pre-vaccination. Our study did not reveal blunting of the infant immune response following the booster dose when comparing antibody levels in infants of mothers with high antibody levels to those in infants of mothers with low antibody levels.

Pertussis toxin is included in all aP vaccines and is considered the most important antigen for which vaccines can induce protection against severe pertussis [21]. Antibodies against other pertussis antigens may also contribute to protection, especially against PRN [18]. However, with the evolution of PRN-deficient *B. pertussis* strains without corresponding loss of vaccine effectiveness, new questions arise regarding the role of antibodies against PRN in the protection against pertussis [22]. Although it is not established what level of antibodies provide protection against pertussis, previous studies have indicated that anti-PT IgG levels below 5 IU/mL are likely non-protective [18,19,23]. A European serosurvey in adults aged 40 to 59 years revealed that when using a cut-off of 0.85 IU/mL, 2% of the Norwegians in this age group had undetectable levels of anti-PT [24]. The corresponding proportion among the pregnant women in our study using this low cut-off is 16% (data not shown), indicating that pregnant women may be more susceptible to infection than middle-aged adults. This is concerning, given that mothers are among the main sources of infection for pertussis in infants [25,26]. Thus, limiting maternal susceptibility to pertussis is of considerable significance.

As newborn infants are protected mainly by transferred maternal antibodies, they may need higher levels of antibodies to prevent infection than vaccinated individuals where protection may also be conferred by cellular responses and immunological memory. The first infant vaccine dose given at 6 to 12 weeks of age world-wide provides partial protection against hospitalisation due to pertussis, although at least two vaccine doses are needed to develop protection in the first year of life [27]. Vaccinating pregnant women against pertussis to protect their infants has proven to be a safe, feasible and effective strategy and demonstrates that transferred maternal antibodies can prevent infection in infants during their first months of life [28]. The estimated effectiveness of maternal vaccination during the second or third trimester is between 69% and 93% against laboratory-confirmed infant pertussis during the first 2 to 3 months of life, and even higher against hospitalisation (91–94%) and mortality (95%) in high-income countries [8]. Our study indicates a low level of protection and high susceptibility for pertussis in infants even when mothers had adhered to adult booster vaccine recommendations. This highlights the importance of vaccination against pertussis during pregnancy to achieve protection in infants, as antibodies wane rapidly in mothers [29].

Vaccinating pregnant women against pertussis has been implemented in an increasing number of countries [5,30]. However, obtaining a high vaccination coverage among pregnant women remains a challenge, with countries such as Czechia, Slovenia and Romania reporting coverage rates below 10% while others struggle to achieve even 50% [5]. High coverage is crucial to obtain the desired effect on disease prevention and public health goals. Research demonstrating that recent pre-pregnancy vaccines may not provide sufficient protection against pertussis in infants as suggested by this study, can be used in maternal vaccine communication to underscore the importance of vaccination in pregnancy and strengthen recommendations.

Vaccine acceptance in pregnancy may be influenced by sociodemographic factors, as a recent Norwegian study has demonstrated [31]. Although our study was conducted at a single centre in Oslo, Oslo University hospital is a public hospital with the highest number of annual births in Norway and the socioeconomic situation of mothers in the catchment area is diverse and includes mothers across the entire socioeconomic range. The mothers in our study had higher vaccination rates for influenza and COVID-19 vaccines than that demonstrated by Hansen et. al. [31], suggesting inclusion of mothers with higher socioeconomic status, but we did not collect socioeconomic data to confirm this. However, data from the literature do not suggest any major impact of socioeconomic factors on antibody levels in vaccinated individuals [32].

Interference of infant vaccine responses from high levels of maternal antibodies, called blunting, has raised concern that maternal vaccination may postpone the disease from early infancy to later in childhood. The focus has been mainly on vaccine responses to pertussis, but also polio, diphtheria and conjugate vaccine antigens using diphtheria carrier proteins, such as pneumococcal vaccines [10-12,33-35]. However, the clinical relevance remains unclear, and the blunting effect seems to be more evident after the primary series [13]. We did not demonstrate any blunted immune responses among the available 14 infant blood samples born by the 18 mothers with high anti-PT above 40 IU/mL, although the number of samples to identify such an effect were limited. This may also be attributed to the fact that we collected samples solely after the 12-month booster, rather than following the primary vaccination series. However, our results are in line with other studies demonstrating that any blunting effect is less pronounced after the first booster [13].

Strengths of our study include a large sample size for a serologic study in pregnant women and their infants, several sampling time points in the same dyads, and an extended follow-up period including samples after the 12-month booster in a 2 + 1 infant vaccination schedule. Health registry data for vaccination in mothers from infancy to adulthood is also unique and provides high quality data on vaccine type and timing of vaccination. With accurate vaccination data from the immunisation registry, we demonstrate that despite high adherence to childhood immunisation and receipt of adult boosters, pertussis antibody levels in mothers are low. Infants alike had high adherence to the infant immunisation schedule and received their hexavalent vaccines in a timely manner, even during the COVID-19 pandemic, and at routine consultations in municipal child health.

Limitations include that data were collected during the COVID-19 pandemic, when infection prevention measures limited the spread of communicable diseases, including pertussis [5]. This may have contributed to the high proportion of mothers with low anti-PT IgG levels. However, the proportion is similar to that found in other studies [29,36,37]. In addition, Norway has a high notified incidence of pertussis, ranging between 37 and 48 cases per 100,000 population in the 5 years before the COVID-19 pandemic, which indicates that although pertussis numbers were low during the pandemic, exposure in pre-pandemic years was common. Moreover, our study was underpowered to identify blunting of the children’s vaccine response after the booster dose at 12 months of age as the number of mothers with anti-PT > 40 IU/mL was only 18, and we did not collect blood samples after the primary vaccination series where blunting has been observed in other studies. We cannot rule out a temporal blunting effect from 3 to 12 months and having additional samples from mothers at delivery and infants after the primary series could have added value to the study. However, additional sample collection was not prioritised due to limited resources. Despite numerous reminders and several meetings with the maternity department, cord blood samples were often forgotten, and therefore only obtained from 53.6% of participants. Conducting the study during the COVID-19 pandemic also complicated compliance to the planned study visits and we achieved full sample sets from only 27% of mother-infant pairs. The completeness of our data would also have improved if all final study visits occurred within the recommended time frame after the 12-month booster, as a higher number of samples would likely have strengthened the blunting analysis. Nevertheless, our study is still large compared with others exploring the susceptibility for pertussis in maternal-infant pairs. Mothers in our study demonstrated high vaccine willingness, many having received influenza and COVID-vaccines as well as adult Tdap-IPV boosters. These mothers are most likely familiar and more up to date with current vaccine recommendations and thus may not be representative for the pregnant population in Norway. However, we still report low levels of antibodies against pertussis in the women included in our study. This may indicate that the susceptibility to pertussis is even higher in the general pregnant population than our results suggest.

## Conclusion

Our study highlights the shortcomings of a childhood immunisation programme in protecting infants in the most vulnerable age against pertussis. We demonstrate a large proportion of women in late pregnancy with low levels of antibodies against pertussis, particularly against PT, thus leaving infants at high risk of severe pertussis disease should they be infected. The results from this study support maternal pertussis vaccination as means to close the susceptibility gap in infants too young to vaccinate and were significant to underpin the recommended introduction of maternal pertussis vaccination in Norway in 2024.

## Data Availability

Data are not publicly available due to legal constraints but may be shared anonymised as baseline dataset upon reasonable request to the authors.

## Supplementary Data

1718000 Supplementary Material
