# Supplementary material for: Maternal and infant immunity against Bordetella pertussis, Norway, 2020 to 2023
Source: Euro Surveill. 2025 Dec 18;30(50):2500450. doi: 10.2807/1560-7917.ES.2025.30.50.2500450 (PMC12719935; doi:10.2807/1560-7917.ES.2025.30.50.2500450)

This supplementary material is hosted by *Eurosurveillance* as supporting information alongside the article

Maternal and infant immunity against *Bordetella pertussis*, Norway, 2020 to 2023

on behalf of the authors, who remain responsible for the accuracy and appropriateness of the content. The same standards for ethics, copyright, attributions and permissions as for the article apply. Supplements are not edited by *Eurosurveillance* and the journal is not responsible for the maintenance of any links or email addresses provided therein.

Supplementary Figure S1: Incidence rate (3-month moving average) for reported cases of pertussis per 100.000 in total population (blue line) and children < 1 year old (red line) for the period 2015-2024.
Data source: Norwegian Surveillance System for Communicable Diseases (MSIS).

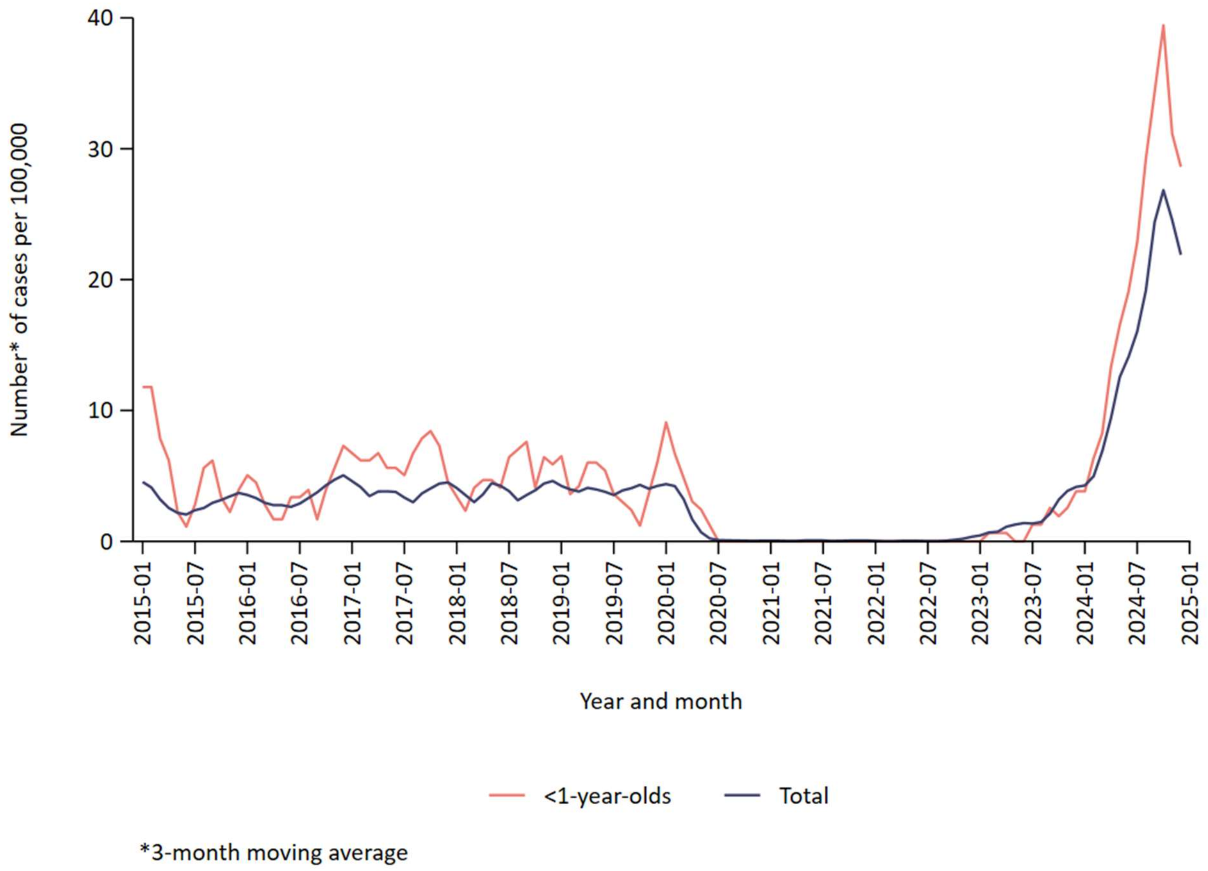

Supplementary Table S1: Antibody levels against pertussis, diphteria, and tetanus related to anti-PT* levels in maternal pregnancy samples, N = 363.

**Mothers' IgG at S1***	**Anti-PRN* Median (IQR)**	**Anti-FHA* Median (IQR)**	**Anti-DT* Median (IQR)**	**Anti-TT* Median (IQR)**
**Anti-PT < 5 IU/ml (n = 174)**	21.5 (6.2 – 81.2)	11.9 (5.9 – 24.6)	0.13 (0.05 – 0.35)	0.99 (0.53 – 1.75)
**Anti-PT 5 – 40 IU/ml (n = 171)**	106.2 (36.2 – 201.3)	38.6 (20.9 – 60.4)	0.17 (0.08 – 0.39)	1.21 (0.71 – 2.00)
**Anti-PT > 40 IU/ml (n = 18)**	231.3 (113.2 – 416.7)	48.1 (35.5 – 112.6)	0.23 (0.07 – 0.71)	1.53 (1.05 – 2.86)

*PT, pertussis toxin; PRN, pertactin; FHA, filamentous haemagglutinin; DT, diphtheria toxoid; TT, tetanus toxoid.
S1 = study visit 1 in late pregnancy.

Supplementary Figure S2: Anti-PT IgG in mothers (N = 363) with corresponding IgG-levels against other antigens, log transformed. a) anti-PRN, b) anti-FHA, c) anti-DT, d) anti-TT. Vertical, dashed line indicates anti-PT IgG = 5, log transformed. Horizontal dotted line indicates anti-DT IgG = 0.015, log transformed (graph c) and anti-TT = 0.1, log transformed (graph d).

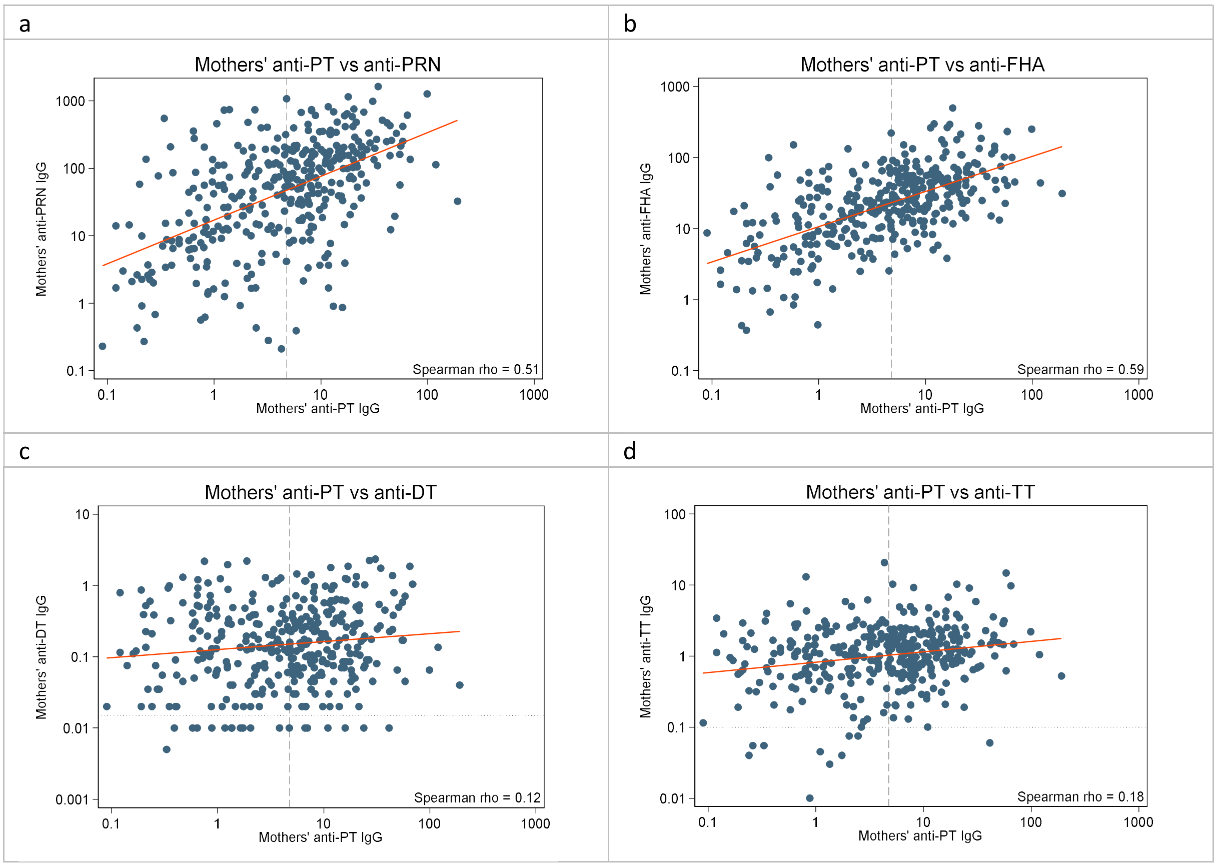

Supplementary Figure S3: Median anti-PT IgG-levels and interquartile range in IU/ml by maternal age, parity and infants' birthweight (BW). Red, dashed line indicates Anti-PT IgG = 5 IU/ml.

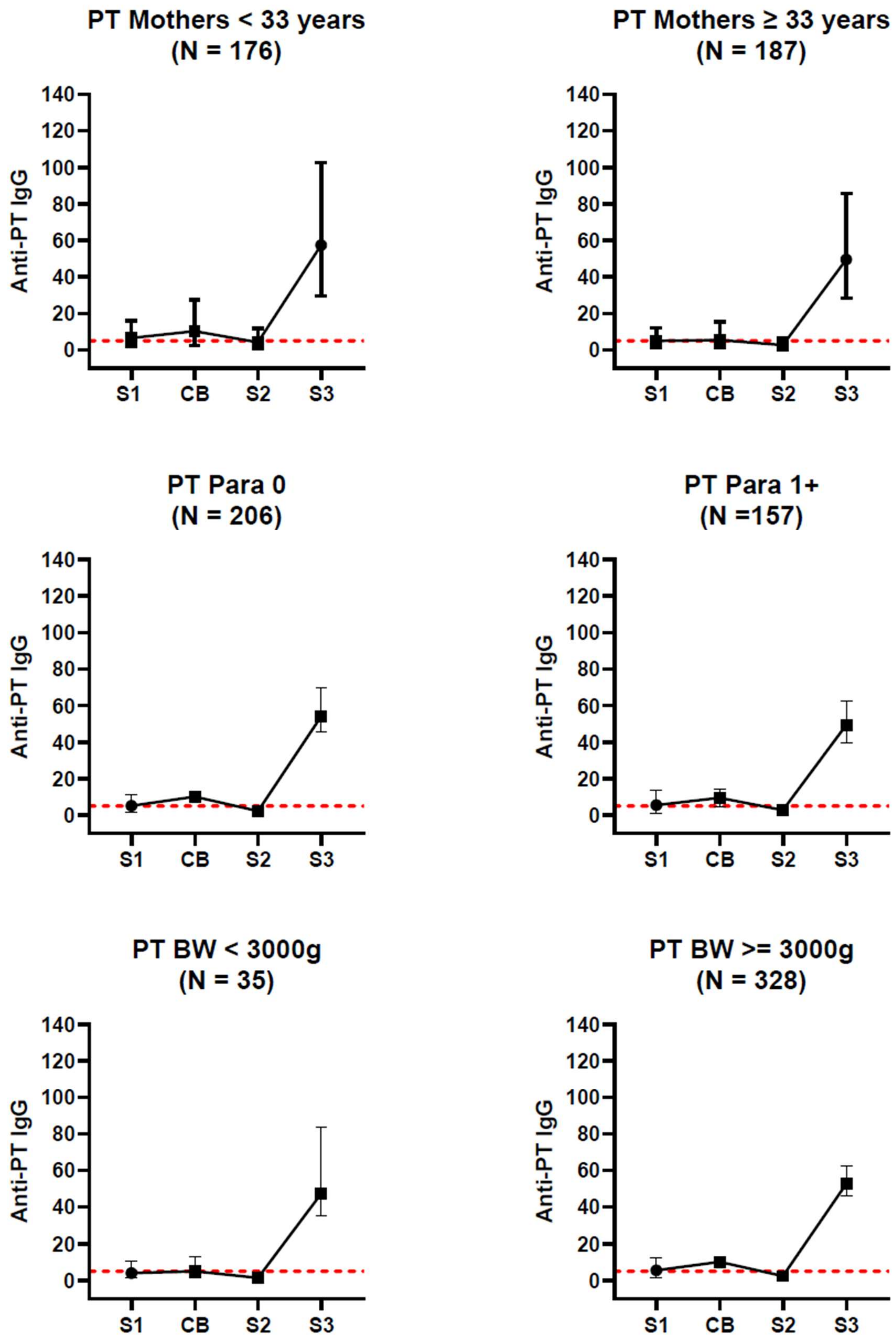

Supplementary Table S2: Placental antibody transfer ratio (cord blood antibodies divided by maternal antibodies in late pregnancy), N = 195.

**Placental antibody transfer ratio**<br>**N = 195**	**CB/S1* ratio**<br>**Median (IQR)**
**Pertussis toxin (PT)**	1.82 (1.44 – 2.05)
**Diphtheria toxin (DT)**	1.50 (1.24 – 1.84)
**Tetanus toxin (TT)**	1.65 (1.36 – 1.98)
**Pertactin (PRN)**	1.58 (1.31 – 1.94)
**Filamentous haemagglutinin (FHA)**	1.68 (1.35 – 2.05)

*CB, cordblood; S1, study visit 1 – mother in late pregnancy.

Supplementary Figure S4: Anti-PT IgG in infants prior to vaccination at 2-3 months of age (study visit 2) (N = 215) with corresponding IgG-levels against other antigens, log transformed. a) anti-PRN, b) anti-FHA, c) anti-DT, d) anti-TT. Vertical, dashed line indicates anti-PT IgG = 5, log transformed. Horizontal dotted line indicates anti-DT IgG = 0.015, log transformed (graph c) and anti-TT = 0.1, log transformed (graph d).

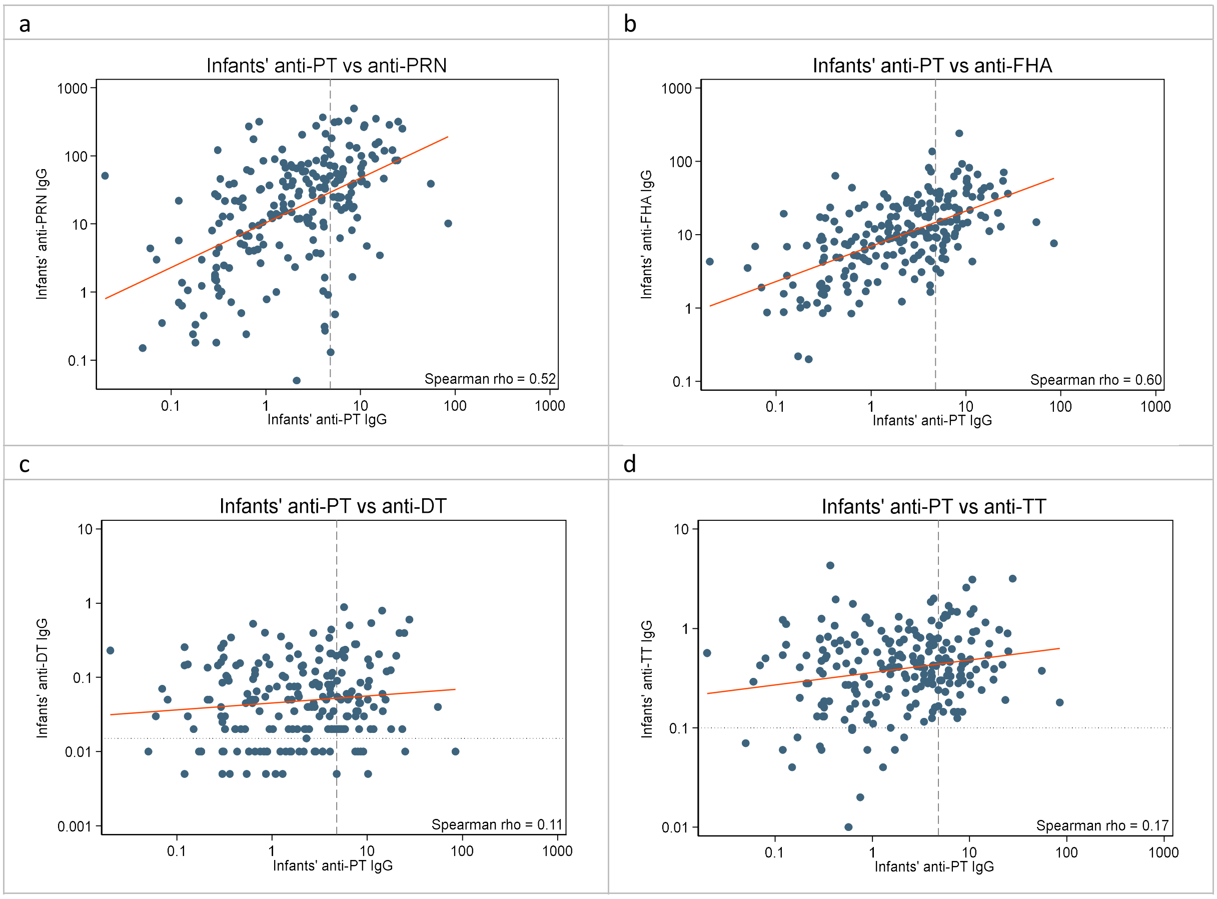

Supplementary Figure S5: Median IgG antibody levels (IU/ml) with IQR for PT at last sampling time, grouped by timing in days of sample after toddler booster dose of aP-containing hexavalent vaccine. Statistical significance indicated by * (P < 0.01).

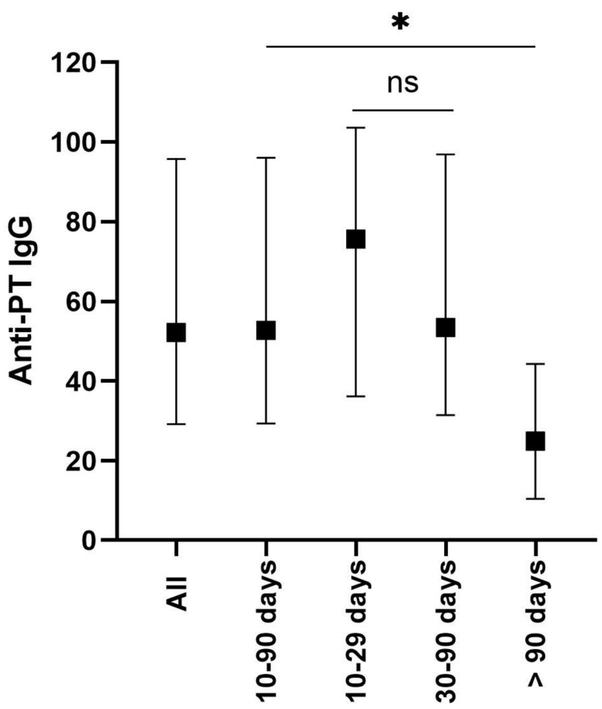

Supplementary Figure S6: Child anti-PT IgG in IU/ml according to interval between 12-month booster receipt and sampling time in days. Horizontal dotted line indicates 10 and 90 days after 12-month booster, respectively. Orange dots represent the three children with anti-PT <5 IU/ml.

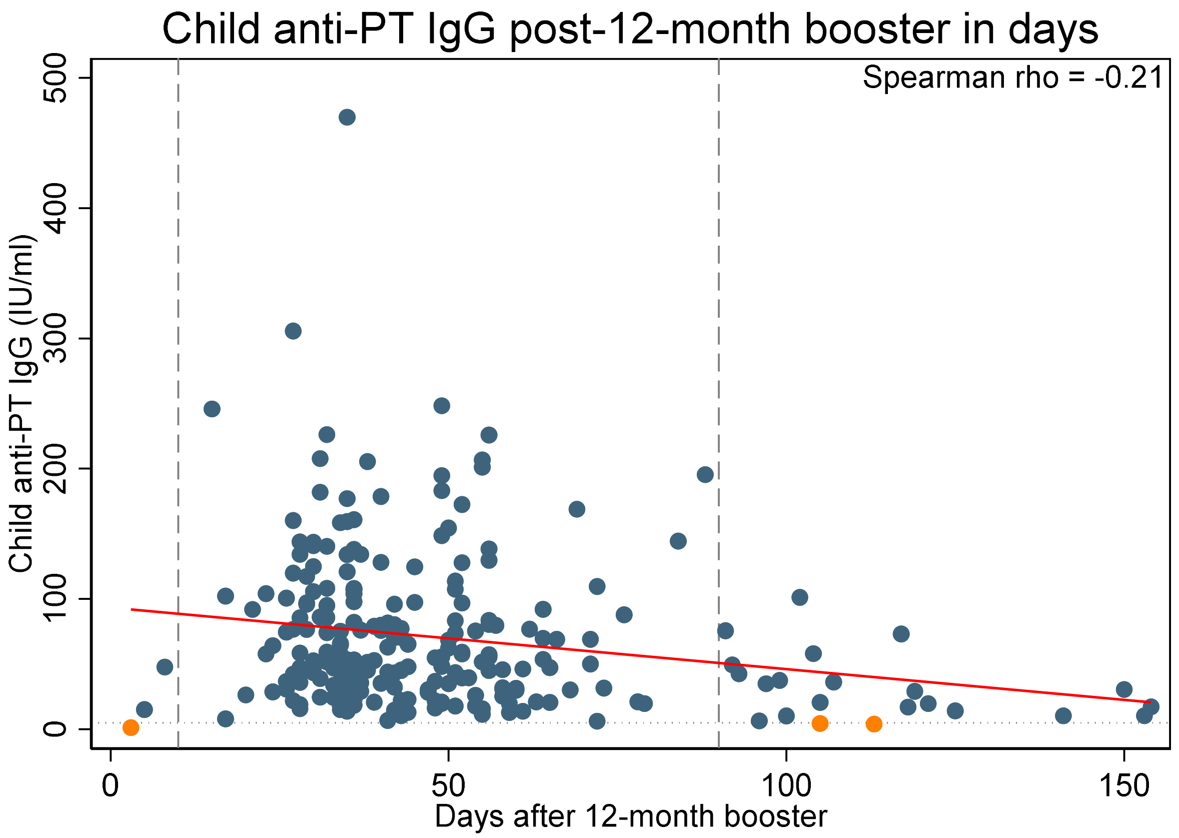

Supplementary Table S3: Blunting analysis comparing infants' antibody levels after the booster vaccine (S3) grouped by maternal antibody levels in pregnancy (S1), restricting to pairs where S3 was collected 10-90 days after hexavalent booster vaccine (N=215 pairs).

N = 215 pairs	Maternal IgG-levels	Infant IgG levels<br>Study visit 3<br>10-90 days after third vaccine dose<br>(Median, IQR)	P-value*<br>(compared to<br>low maternal group)
**PT**	Low: < 5 IU/ml<br>n = 93	52.8 (31.6–97.4)	-
	Intermediate: 5 – 40 IU/ml<br>n = 108	63.2 (32.9-99.3)	0.66
	High: > 40 IU/ml<br>n = 14	49.3 (28.7–79.8)	0.76
**DT**	Low: < 0.015 IU/ml<br>n = 10	2.48 (1.94–3.87)	-
	Intermediate: 0.015-1.0 IU/ml<br>n = 187	2.74 (1.54-4.15)	0.93
	High: > 1.0 IU/ml<br>n = 18	2.03 (1.45–2.66)	0.36
**TT**	Low: < 0.1 IU/ml<br>n = 3	2.57 (2.18 – 4.59)	-
	Intermediate: 0.1-1.0 IU/ml<br>n = 90	2.14 (1.37-4.07)	0.43
	High: > 1.0 IU/ml<br>n = 122	2.63 (1.19–4.83)	0.80

*Statistical test: Mann–Whitney U test

Supplementary Figure S7: Mothers' anti-PT IgG levels, log transformed, with a) time in years since last aP before pregnancy start (n=212) and b) calendar date for sampling (n=363). Dotted line in grey indicates anti-PT = 5 IU/ml.

a)

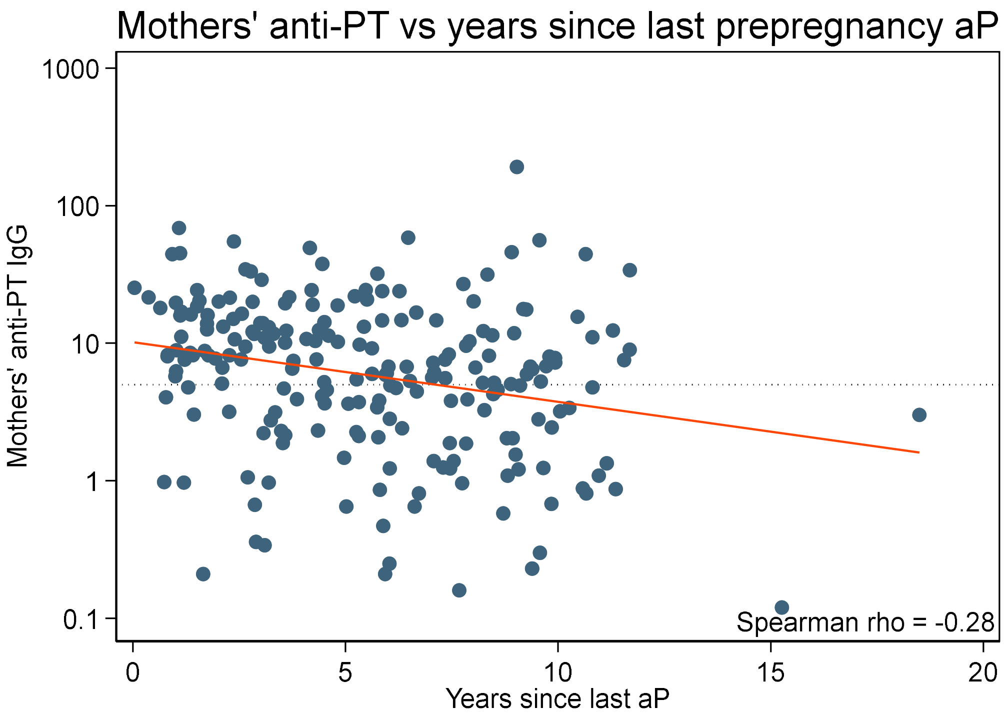

b)

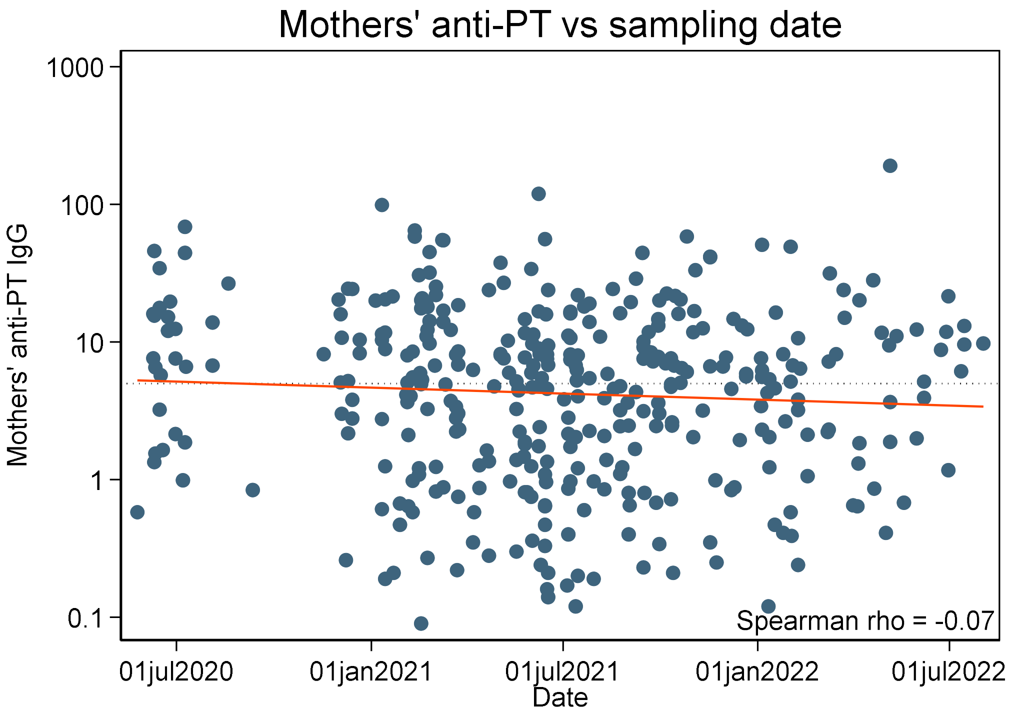

Supplement: Supplementary Material [file 25-00450_GREVE-ISDAHL_Supplement.pdf]
